# Perineal burn injury with hidradenitis suppurativa: A case report

**DOI:** 10.1097/MD.0000000000032525

**Published:** 2022-12-23

**Authors:** Linyue Wang, Wenyue Qi, Jihua Gao, Maosheng Tian, Hongyuan Sun, Wencong Xu

**Affiliations:** a Graduate School, Hebei University of Chinese Medicine, Shijiazhuang, China; b Anorectal Department, The First Affiliated Hospital of Hebei University of Chinese Medicine, Shijiazhuang, China; c Key Laboratory of Integrated Chinese Medicine and Western Medicine for Gastroenterology Research (Hebei), Shijiazhuang, China.

**Keywords:** apocrine glands, burn injury, case report, hidradenitis suppurativa

## Abstract

Hidradenitis suppurativa (HS) is a chronic, inflammatory skin disease which usually presents with painful, deep-seated, inflamed lesions in the apocrine gland-bearing areas. Severe HS has interconnecting sinus tracts and when it occurs in the perianal region, perianal fistulas can form. Obesity and smoking are 2 risk factors for the development of HS.

**Patient concerns:**

We report a case of a 33-year-old male diagnosed with perianal HS and perianal fistula following a burn injury to the area that occurred during childhood.

**Diagnosis:**

Through integration of the clinical signs and imaging results, the patient was diagnosed with HS, Hurley stage III with perianal fistulas.

**Interventions:**

The patient accepted surgical therapy. Performed under the general anesthesia, the procedure comprised sinus tracts excision and drainage.

**Outcomes:**

The patient was discharged from the hospital 6 weeks after surgery.

**Lessons:**

The pathogenesis of the HS in this case was the burn injury interfering with sweat gland formation around the anus. Moreover, the scar from the burn made surgical treatment difficult.

## 1. Introduction

Hidradenitis suppurativa (HS) is a chronic, inflammatory skin disease, which usually presents after puberty with painful, deep-seated, inflamed lesions in the apocrine gland-bearing areas.^[1]^ Approximately 19% of HS occurs in the perianal region.^[2]^ The true incidence of HS is unknown, but prevalence estimates vary from 0.00033% to 4.1%.^[3]^ The disease can have a greatly negative effect on the quality of life of affected patients, depending on disease severity.^[4,5]^

## 2. Case report

**Figure 1.**
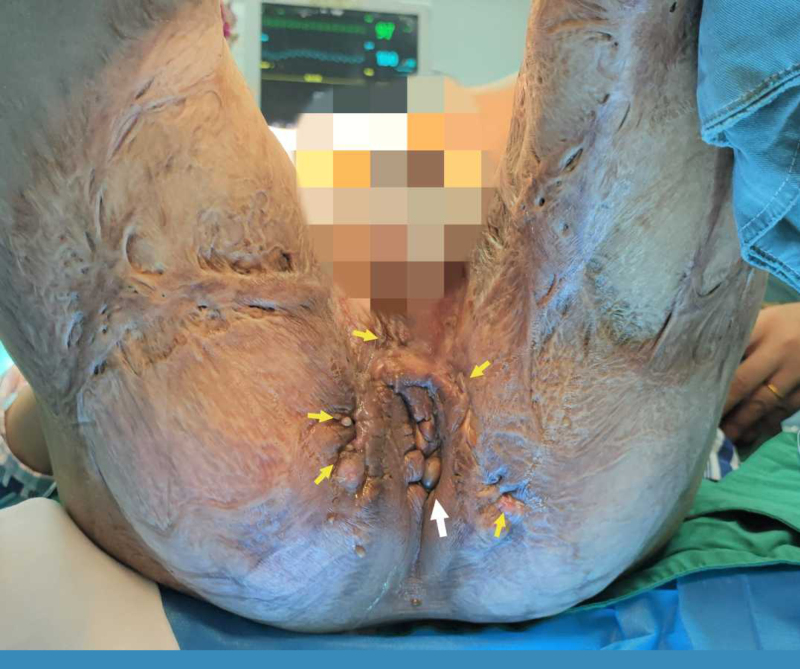
Sinus tracts (yellow arrows) and anus (white arrow).

The patient, a 33-year-old male, was hospitalized for recurrent perianal suppuration. An accident when he was 3 years old led to a burn injury involving both thighs, buttocks, and the perineum. A year prior to this case report’s presentation, recurrent abscesses formed around the anus and subsequently ruptured and drained. The suppurative lesions became painful 2 weeks before admission. Physical examination showed that extensive scar tissue had formed on the buttocks, perineum, and back and inner surfaces of the thighs, which impaired the hip abduction. In addition, a few dense comedones could be seen on the back of the thighs. Several draining fistulas with purulent secretions were located in the perianal area (Fig. 1), and there were inflammatory granulomas at the openings of the tracts.

Blood tests showed a high neutrophilic granulocyte percentage (75%), increased leukocytes (10.74 × 10^9^/L) and low erythrocytes (3.88 × 10^12^/L) and hemoglobin (114g/L). Platelet count was 473 × 10^9^/L. The pelvic MRI showed a small, bar-shaped area of high T2 signal at the 12 o’clock position of the anal canal and patchy, long T2 signal bilaterally in the intersphincteric zone. The branches of the latter signal reached the subcutaneous tissue, with high signal intensity on diffusion-weighted images. The posterior of the sacrum and the natal cleft had an irregular, long T2 signal. A 3.8 cm × 3.0 cm area of nonhomogeneous echogenicity was found at the sacrococcygeal region by ultrasonography.

Through integration of the clinical signs and imaging results, the patient was diagnosed with HS, Hurley stage III with perianal fistulas. In this case, surgical intervention was the best chance of cure.^[6]^ Performed under the general anesthesia, the procedure comprised sinus tracts excision and drainage. After surgery, the wound dressing was changed every day. The patient was discharged from the hospital 6 weeks after surgery.

## 3. Discussion

Obesity and smoking are 2 known risk factors for the development of HS.^[2]^ In contrast with the average HS patient, this patient was a nonsmoker with a lean figure and should not susceptible to HS. In this case, the trigger was likely the burn injury. First, in the hypertrophic burn scar, the secretory part of the sweat gland had changed anatomic lotion and was found to be expanded and organized irregularly.^[7]^ Abnormal sweat glands formed the morphological basis for pathological development of HS. Second, a burn patient shows a complexity of inflammatory response reactions.^[8]^ There was a study showed that burn injury induces a persistent innate inflammatory response, releasing immature neutrophils and shifting the T cells to the pro-inflammatory phenotype.^[9]^ A burn wound may suffer from the effects of inflammatory factors for a prolonged period of time, and inflammation provides the environment for HS pathogenesis.^[10]^ For all these reasons, the burn injury was the key factor that induced HS in this patient.

Hurley staging is a common assessment and classification method for HS, with 3 degrees of severity.^[11]^ Hurley I is characterized by abscessation without sinus tract formation. Hurley III has diffuse interconnected tracts. The severity of Hurley II is between Hurley I and III, characterized by limited number of sinuses and/or scarring. This patient had several fistulas with purulent secretion, meeting the diagnosis criteria of HS at Hurley stage III with perianal fistula. Only 1% to 4% of HS patients progress to Hurley III,^[1,6]^ with a significant impact on patients social life.^[12]^ Surgical treatment is indicated in stage III with almost one third of these patients undergoing surgery and topical or systematic antibiotics combined treatment.^[13]^ Excision and deroofing are commonly used by surgeons, while incision and drainage are not recommended because of the risk of recurrence.^[6]^ Interestingly, for this patient, drainage and incision was the better method as the burn scar presented difficulties in successful shifting or healing of the skin, and the wound healing would have been slower than in other patients.

Recent studies have found that biologicals have a satisfactory effect in moderate-to-severe HS. Adalimumab, a tumor necrosis factor inhibitor, was efficacious when used in conjunction with wide-margin excision surgery.^[14]^ Furthermore, early adalimumab use could expedite the response time of HS patients.^[15]^ Another study showed the use of an interleukin-17 inhibitor, bimekizumab, led to more clinically meaningful improvements than adalimumab in a phase 2, double-blind, placebo-controlled randomized clinical trial.^[16]^ It is clear that biologicals are useful in treating HS, but due to objective constraints, only classic treatments were applied in this case.

In conclusion, in this case of severe HS caused by burn injury, the chosen therapy differed from that usually applied to HS patients. For perianal burn injury patients, the recovery of apocrine glands around the anus should be taken into consideration in order to avoid the secondary, related diseases like HS. Once there is diagnosis of perianal HS, early use of a biological may be beneficial.

## Acknowledgments

We thank the Enago (https://www.enago.cn/) for its linguistic assistance during the preparation of this manuscript.

## Author contributions

**Conceptualization:** Linyue Wang.

**Investigation:** Maosheng Tian.

**Methodology:** Wenyue Qi.

**Resources:** Jihua Gao.

**Supervision:** Jihua Gao.

**Visualization:** Hongyuan Sun, Wencong Xu.

**Writing – original draft:** Linyue Wang.

**Writing – review & editing:** Jihua Gao.
